# Acknowledgment to Reviewers of *Bioengineering* in 2019

**DOI:** 10.3390/bioengineering7010011

**Published:** 2020-01-16

**Authors:** Bioengineering Editorial Office

**Affiliations:** MDPI, St. Alban-Anlage 66, 4052 Basel, Switzerland

The editorial team greatly appreciates the reviewers who have dedicated their considerable time and expertise to the journal’s rigorous editorial process over the past 12 months, regardless of whether the papers are finally published or not. In 2019, a total of 112 papers were published in the journal, with a median time to first decision of 15 days and a median time from submission to publication of 38 days. The editors would like to express their sincere gratitude to the following reviewers for their generous contribution in 2019:
Acquah, CalebLin, WeiAgarwal, GunjanLin, ZhiweiAghayi, EmadLiu, AipingAhearne, MarkLiu, LimingAhmad, KhurshidLoessner, DanielaAiello, OrazioLukaszewicz, MarcinAkhtar, RiazM D Almeida, M CatarinaAlbert, SilviaMaffiodo, DanielaAlvarado-Morales, MerlinMallis, PanagiotisÄmmälä, AriMandatori, DomitillaAmoako, KagyaMarrazzo, PasqualeAn, YuMartin, Andrew ReadAngulo, JavierMartindale, Christine F.Anssari-Benam, AfshinMartin-Deleon, Patricia A.Aravalli, Rajagopal N.Martins, GilbertoArdelean, Lavinia CosminaMaruthamuthu, MuraliArunkumar, AbhiramMatwijczuk, ArkadiuszAshry, MohamedMayhan, William G.Awtoniuk, MichałMcGuigan, APAyala-Orozco, CiceronMeng, FanbenAzman, SametMikhaylin, SergeyBadea, MihaelaMilcovich, GesmiBalogh-Weiser, DianaMitchell, AndrewBaran, JaroslawMobed-Miremadi, MaryamBauer, Denis C.Modine, ThomasBaumgarten, ThomasMontañez, RaúlBen Arfa, BasamMoradshahi, MehradBhattarai, ArjunMorrison, MelanieBilchick, Kenneth CMosig, Alexander S.Bitra, ArunaMou, YongchaoBlaser, MarkMowla, AlirezaBorman, PhilMozumder, Md. Salatul IslamBorrero-de Acuña, Jose ManuelMueller, MischaBranch, Matthew JamesMukhopadhyay, SubhradipCaimmi, PhilippeMuntimadugu, EameemaCalandrini, SaraMuszyński, SiemowitCarotenuto, ClaudiaMutreja, IshaCastro, AndreMyung, JaewookCerrone, FedericoNajdahmadi, AvidChan, Yau KeiNarayanan, BarathChang, MarvinNarkar, AmeyaChen, H. IsaacNashimoto, YujiChen, HuizhiNethi, Susheel KumarChen, Yu-ChihNguyen, HongChiulan, IoanaNguyen, TrieuChoi, DonghoNieto, DanielChoo, Pei LingNivedita, NiveditaChua, Chee KaiNucci, NathanielChwalek, KarolinaObruca, StanislavChybowski, Leszek MarekOchia, RuthCiesielski, SlawomirOlaru, MihaelaContessi Negrini, NicolaOmata, SeijiCorrigan, NathanielOnofrillo, CarmineCostanzo, SalvatoreOsyczka, Anna MariaDe Candia, PaolaPal, Ajay De Eugenio, Laura IsabelPanico, AntonioDehghan, AmirPark, Soo-JinDel Cerro Sánchez, CarlosPasta, SalvatoreDel Gaudio, CostantinoPatnaik, SouravD’Errico, GerardinoPaula, Anabela BaptistaDeshpande, Gauravi Peer, PetraDi Ruberto, CeciliaPenchev, HristoDiogo, MargaridaPitcher, DavidDo, Minh TruongPizzolanti, GiuseppeDo, Quoc CuongPlacido, JerssonDoddasomayajula, RaviPolini, AlessandroDöllinger, MichaelPowell, KimerlyDoustkhah Heragh, EsmaeilPrados-Privado, MaríaDrapaca, CorinaProt, VictorienDubey, ShriPulidindi, Indra NeelEiró, NoemíPulliam, LynnElreedy, AhmedPuniya, Bhanwar LalErgene, CansuRaju, Arun Satheesh KumarErtas, AtilaRanjan, AtulEsmaeili Pourfarhangi, KamyarRanjit, SumanEspino, Daniel M.Rausch, ManuelAzhagiya Singam, E.R.Riedel, Sebastian L.Facco, PierantonioRocha-Martin, JavierFang, Hsu-WeiRodríguez-Contreras, AlejandraFare, SilviaRom, SlavaFarrugia, BrookeRowland, TeishaFarzaneh, VahidRuddock, Lloyd W.Feng, HuichengRugonyi, SandraFeng, LuSaha, Amit KumarFernandez-Morales, Francisco JesusSalehi-Reyhani, AliFerrigno, AndreaSalman, MootazFissell, WilliamSalmina, AllaFrances, ChristineSamal, SangramFrank, StefanieSanthanam, AbiramiFriedenberg, DavidSanz-Garcia, AndresFue, KadegheSavelyeva, NataliaGaffet, EricSchaiquevich, PaulaGalla, Hans-JoachimSchilsky, MichaelGao, XinSchwaminger, SebastianGarofalo, MariangelaSedlacek, PetrGerding, HeinrichSekine, MasashiGizdavic-Nikolaidis, MarijaSerrancolí, GilGluck, JessicaSerwotka-Suszczak, AnnaGomez Cerezo, Maria NatividadSharbeen, GeorgeGómez, AlbertoSheikh, Søren PaludanGonzález-Aseguinolaza, GloriaShelke, GaneshGrãos, MárioShevchuk, AndrewGrubitzsch, HerkoShi, JieGuglielmino, SalvatoreShimizu, KazuyukiGundapaneni, DineshShrestha, NamitaGupta, KuldeepSingh, AnupGursoy, DogaSingh, RaghuveerHara, Emilio SatoshiSinghal, NareshHara, HirofumiSivathanu, VivekHasan, WohaibSolitro, GiovanniHaverkamp, Richard G.Soran, Z. OzlemHe, MingyuSrinivasan, SwethaHeim, FredericSriram, GopuHeindryckx, FemkeStachewicz, UrszulaHerrgen, LeahStrauss, SarahHilgen, GerritStrohbach, AnneHinman, SamSung, Tzu-ChengHo, Allen L.Suryadevara, VidyaniHochstetter, AxelSuwal, ShyamHolnthoner, WolfgangSydlowski, LukaszHorbett, Thomas ASzweda, RozaHorton, Edward RoyTaglietti, AngeloHu, JingjieTam, Roger Y.Hu, WeichihTan, Amy G.Y.Huang, ChenyuTan, LayHuling, JenniferTaniguchi, HironariIconaru, Simona LilianaTanner, Julian AlexanderIjima, HiroyukiTatullo, MarcoInfante, ArantzaTepper, GaryIngrassia, TommasoTkáč, JánIraci, NunzioToh, Wei SeongIrusta, UnaiToma, MilanIstván, HornyákToya, YoshihiroJahanipour, JahandarTrindade, AlexandreJammalamadaka, UdayabhanuTruong, DanhJeison, DavidTsai, Ang-ChenJewell, Mark LTsubaki, ShuntaroJoddar, BinataTsuboko, YusukeJohnson, ChristopherTurhanen, PetriJørgensen, BodilValente, JoanaKadem, LyesValenti, AnnaKaehr, BryanValeriani, DavideKaizer, Marina Da RosaVan Wyk, NielKalyana Sundaram, Ramalingam VenkatVanderpool, Rebecca R.Kantak, ChaitanyaVattepu, RaviKaraś, MagdalenaVaz, Maria De Fátima ReisKhan, IrfanVecchione, Dr RaffaeleKhetani, Salman RVerbeeck, KristofKido, JunVerstegen, Monique M.A.Kim, Beom SooVitali, AndreaKim, Hong KyunVollmer, MarcusKim, JeonghunVolova, TGKim, JihoonVon Euw, StanislasKim, Kyoung-YeolWang, ShuangyiKim, SejoongWang, ShuolunKimura, HidehitoWang, XiaojuKimura, HiroshiWedde, SeverinKoivuniemi, AndrewWidyaya, Vania TandaKorupalli, ChiranjeeviWitek, LukaszKourmentza, ConstantinaWitowski, JanKourmentza, KonstantinaWoelfl, StefanKouw, Wouter M.Wolanśki, WojciechKovanecz, IstvanWoldemariam, Daniel MiniluKruithof, BoudewijnWorkman, VictoriaKrzyżewski, Roger MYadav, PramodKucharska, KarolinaYan, JipengKumar, AnujYang, Seung-HoKuvshinov, DmitriyYannarelli, GustavoLand, RüdigerYasmin, YasminLanglois, ValerieYazdi, ImanLauschke, Volker M.Yepuri, Nageshwar RaoLeavesley, DavidYip, Bon HamLee, EricYong, Kar WeyLemma, EnricoYu, JiayiLeonetti, AlessandroYu, NaLeppiniemi, JenniZacharof, Myrto-PanagiotaLi, FanghuaZaharia, CatalinLi, FengZeng, JILi, HaijunZeng, WeiLi, XiangZhang, RunLiang, C.Zhang, YuLiang, JunZhou, WeijieLim, JasonZhu, XiaoliangLim, Khoon


